# Examining B-cell dynamics and responsiveness in different inflammatory milieus using an agent-based model

**DOI:** 10.1371/journal.pcbi.1011776

**Published:** 2024-01-23

**Authors:** Bryan Shin, Gary An, R. Chase Cockrell

**Affiliations:** Department of Surgery, University of Vermont Larner College of Medicine, Burlington, Vermont, United States of America; University of Pittsburgh, UNITED STATES

## Abstract

**Introduction:**

B-cells are essential components of the immune system that neutralize infectious agents through the generation of antigen-specific antibodies and through the phagocytic functions of naïve and memory B-cells. However, the B-cell response can become compromised by a variety of conditions that alter the overall inflammatory milieu, be that due to substantial, acute insults as seen in sepsis, or due to those that produce low-level, smoldering background inflammation such as diabetes, obesity, or advanced age. This B-cell dysfunction, mediated by the inflammatory cytokines Interleukin-6 (IL-6) and Tumor Necrosis Factor-alpha (TNF-α), increases the susceptibility of late-stage sepsis patients to nosocomial infections and increases the incidence or severity of recurrent infections, such as SARS-CoV-2, in those with chronic conditions. We propose that modeling B-cell dynamics can aid the investigation of their responses to different levels and patterns of systemic inflammation.

**Methods:**

The B-cell Immunity Agent-based Model (BCIABM) was developed by integrating knowledge regarding naïve B-cells, short-lived plasma cells, long-lived plasma cells, memory B-cells, and regulatory B-cells, along with their various differentiation pathways and cytokines/mediators. The BCIABM was calibrated to reflect physiologic behaviors in response to: 1) mild antigen stimuli expected to result in immune sensitization through the generation of effective immune memory, and 2) severe antigen challenges representing the acute substantial inflammation seen during sepsis, previously documented in studies on B-cell behavior in septic patients. Once calibrated, the BCIABM was used to simulate the B-cell response to repeat antigen stimuli during states of low, chronic background inflammation, implemented as low background levels of IL-6 and TNF-α often seen in patients with conditions such as diabetes, obesity, or advanced age. The levels of immune responsiveness were evaluated and validated by comparing to a Veteran’s Administration (VA) patient cohort with COVID-19 infection known to have a higher incidence of such comorbidities.

**Results:**

The BCIABM was successfully able to reproduce the expected appropriate development of immune memory to mild antigen exposure, as well as the immunoparalysis seen in septic patients. Simulation experiments then revealed significantly decreased B-cell responsiveness as levels of background chronic inflammation increased, reproducing the different COVID-19 infection data seen in a VA population.

**Conclusion:**

The BCIABM proved useful in dynamically representing known mechanisms of B-cell function and reproduced immune memory responses across a range of different antigen exposures and inflammatory statuses. These results elucidate previous studies demonstrating a similar negative correlation between the B-cell response and background inflammation by positing an established and conserved mechanism that explains B-cell dysfunction across a wide range of phenotypic presentations.

## Author summary

In this work, we present a computational model of immune memory formation in B-cells, the phenomenon that allows a human being to develop immunity against pathogens they have previously encountered. The computational model was developed as an agent-based model, in which cells are represented individually and perform their cellular functions and actions in response to stimuli form the environment and other cells. We examine the process of immune memory formation in the context of sepsis, a highly inflammatory condition that can occur after serious injuries, diseases, or trauma. We then use this model to offer an explanation for recent findings discussing the impact of repeated COVID-19 infection; specifically, we note that the referenced study was performed in a relatively narrow population, those that sought care at a Veterans Affairs (VA) hospital, that would experience higher than normal levels of background inflammation. We use the model to demonstrate that this background inflammation can impair the process of memory formation in response to a COVID-19 infection and posit one explanation for increasing severity of reinfections in the VA population.

## Introduction

The B-cell immune system is a complex system of cells, antibodies, and cytokines that serves to aid in the body’s response to infection by neutralizing infectious agents through the phagocytic actions of naïve and memory B-cells [1]. Furthermore, the B-cell immune system confers immunity to future infection by microorganisms carrying the same antigen through generation of antigen-specific memory B-cells and antibody-secreting plasma cells.

There has been increasing interest in the role of B-cells during sepsis, which is characterized by disordered inflammation that includes excessive pro-inflammation that leads to collateral tissue and organ damage as well as pronounced immune dysfunction that leaves patients susceptible to infection by other opportunistic organisms [2]. This immune impairment contributes heavily to a patient’s failure to recover from the initial septic insult by rendering patients susceptible to nosocomial infections, and both prolongs intensive care unit stays and increases mortality rates [3,4]. Specifically, B-cell immunoparalysis is thought to be a significant contributor to this immunosuppression, as sepsis can prevent the adequate formation of B-cell subtypes that normally would aid in neutralizing an infectious challenge [5,6].

Studies have identified three important mechanisms behind B-cell dysfunction: B-cell apoptosis, anergy, and differentiation into regulatory B-cells [5,6]. The causal factors that govern these mechanisms can be attributed to specific cytokines. Tumor Necrosis Factor-alpha (TNF-α) regulates the induction of apoptosis in all B-cell lineages [7]. Interleukin-10 (IL-10) and Interleukin-6 (IL-6) induce B-cell anergy by downregulating the expression of the activation marker Cluster of Differentiation Receptor 21 (CD21) on naïve and memory B-cells [5]. IL-6 also induces terminal differentiation into regulatory B-cells, which then exert a compounding immunosuppressive effect on the circulating B-cells and other inflammatory cells [8].

While processes that generate a state of sepsis can result from the system’s response to a large acute insult, these same processes remain present in chronic inflammatory conditions such as type 2 diabetes mellitus or obesity [9,10]. In addition, aging populations have also been shown to exist in chronic states of low inflammation, termed “inflammaging”, which may similarly induce B-cell senescence [11,12]. These patients can experience an impaired B-cell responsiveness induced by IL-6 and TNF-α [11,13], similar to that seen during sepsis. This B-cell dysfunction contributes to a higher risk for not only primary infection but also re-infection by bacteria or viruses. For example, a recent study found that patients with diabetes mellitus were more susceptible to re-infections by SARS-CoV-2 due to impaired immune function [14–16].

The range of clinical phenotypes and conditions potentially impacted by impaired B-cell function presents both challenges and opportunities in terms of understanding the dynamics of the B-cell response. Challenges arise from the heterogeneity of factors present in these divergent populations, including differences in the insults and potential pre-existing immune derangements present in each group. However, opportunities arise from the fact that the same basic structure of the B-cell response can generate this wide range of distinct configurations. While experimental biology is able to examine, in detail, the dynamics present in specific disease or intervention models (i.e., acute infection, chronic diseases, vaccine responsiveness, etc.), computational/mathematical modeling can help integrate the hypotheses structures/conceptual models generated from experimental work and evaluate, dynamically, whether such models can recapitulate the features of different types of perturbations. We have termed this process *dynamic knowledge representation*, but this principle unpins the concept of mathematical biology. Towards this end, we and others have proposed the use of a computational modeling method, agent-based modeling, as a means of dynamic knowledge representation [17,18]. Agent-based modeling involves representing complex systems as a population of interacting components, or agents, that are driven by a specified set of rules for local interactions and has been used extensively to study immune responses [19–21], allowing for the creation of dynamic, in-silico models that enable interrogation of the represented variables. An agent-based model (ABM) can serve as a means of dynamic knowledge representation that allows the visualization of complex interactions that can sometimes produce unanticipated and paradoxical results [17]. When properly validated, ABMs can even serve as experimental platforms for simulation experiments that allow for a much larger range of manipulation compared to what would be possible in a laboratory setting and greater precision in observation of cell behaviors. The simulations may reveal new directions for laboratory experiments or act in lieu of interventions that are too complex to perform in the real world.

Modeling the immune system with ABMs represents some of the earliest applications of this method in the biomedical arena, and there have been previous studies that utilize ABMs to study the B-cell immune system in particular. In 1992, Celada and Seiden developed a computer model, the IMMSIM, in the form of a ‘cellular automaton’ [22] which represented discreet cellular entities that progressed through timesteps as a boolean model. It included interactions between B-cells, T-cells, and antigen-presenting cells, as well as antibody generation. However, the cellular automaton did not integrate the spatiotemporal relationship between the interacting cell types in the context of a lymph node follicle. The Celada-Seiden automaton was extended into an agent-based model by Stracquadanio et al. [23], who similarly aimed to study antibody generation and antigen dynamics by integrating the actions of B-cells, plasma cells, antigen-presenting cells, and T-cells. In addition, it incorporated antigen-receptor epitope matching in the form of string matching to study the effects of cross-reactivity. In both models, however, the B-cell subtypes and their differentiation dynamics were not distinguished, and cytokines were not present in the environment. Starting in 1997, a group of researchers expanded the capabilities of cellular automata models of the immune system by introducing the ability to parallelize their execution to take advantage of advances in high performance computing architectures [24], and implemented additional optimizations to allow for simulations of millions of interacting cells [25]. Refinements of this model of the immune system [26] have been used to study, among other processes, the timing of vaccination boosters [27] and immune dynamics arising from SARS-COV2 infection [28]. Other models have studied specific processes within the B-cell response such as affinity maturation within germinal centers, which is a crucial component of potent immune memory formation [29]. These projects utilized mathematical modeling and original differential equations to study the relationship between clonal abundancy and affinity [30], as well as the effect of vaccine timing and antigen dosage on the level of affinity formation [31].

Herein, we describe the B-cell Immunity Agent-based Model (BCIABM), a computational model that incorporates the key steps of B-cell differentiation in response to antigenic stimuli and secondary exposures. We build upon the aforementioned prior modeling projects by describing a broader context of the B-cell response, from antigen inoculation to the formation and subsequent waning of immune memory in the context of systemic inflammation. We implement the differentiation pathways of an expanded set of B-cell subtypes: naïve, germinal center, memory, short-lived and long-lived plasma, and regulatory B-cells. Importantly, we implement the cytokines and mediators described earlier that influence B-cell fates in order to study the effects of concurrent systemic inflammation on the B-cell response. Initial evaluation focused on the ability of the BCIABM to reproduce the dynamics of mild antigen stimuli as well as large insults seen in sepsis, and subsequently used to examine the effect of chronic, baseline low-level inflammation.

## Methods

### Design rationale

This current version of the BCIABM is focused on the generation of B-cell lineages that occurs in the lymph node follicle and includes the following B-cell subtypes: naïve B-cells, short-lived plasma cells (SLPC), long-lived plasma cells (LLPCs), memory B-cells, germinal center B-cells, and regulatory B-cells. Though there are higher granularity categorizations of B-cells, we chose a more generalized subtyping schema to create a broad characterization of B-cell differentiation that can still be useful in studying B-cell behavior. We included the cytokines that were most prevalently described to influence B-cell differentiation fate, as these play a significant role in the extent of antibody and memory generation in response to antigen. In addition, we used the cytokines TNF-α, IL-6, and IL-10 along with their downstream effects on B-cells to represent the features of systemic inflammation, either at transiently higher levels, as would be seen in response to a significant septic insult, or at a lower, continuous levels more akin to chronic disease states.

### Literature review and rule selection

A literature review was carried out to identify key features of the B-cell response and used to construct state diagrams for each of the cell types involved. These components are represented as “agents” in the ABM that behave according to specified rules, and each have a set of inputs and outputs (see Table 1). The state diagram details each agent-type’s function in the immune system and their interactions with other cells and cytokines.

**Table 1:** Cell Types and their Rules.

All Motile Cells	1. All motile cells move with a speed of one grid space per time step. The direction of movement is initially randomized for each cell but can be influenced by surrounding cytokines depending on cell type. 2. All B-cell lineages can undergo TNF-α-induced apoptosis [33]. 3. All B-cell lineages can undergo differentiation into regulatory B-cells, which is induced by IL-6 and IL-21 [34].
Follicular Dendritic Cells (FDCs)	1. Located in the center of the follicle and are completely immobile [35]. 2. Act as the primary antigen-presenting cell of the system. FDCs take up incoming antigen from the lymph node’s subscapular sinuses during inoculation and then display them to B-cells for a prolonged duration to help magnify the B-cell response [36]. 3. Secrete CXCL13, which is a chemokine used to help B-cells localize to the lymph node follicle from the blood [1,35]. 4. Secrete IL-6 [37], which influences the differentiation fate of activated B-cells.
Naïve B-Cells	1. Naïve B-cells enter into the follicle at a constant rate to represent newly matured naïve B-cells from the bone marrow. 2. Chemotaxis is determined by two main chemokines: S1P1 and CXCL13, of which the receptors on the B-cell are S1PR1 and CXCR5, respectively. Both chemokines are implemented as patch variables. a. CXCR5 expression allows the naïve B-cells to localize to the lymph node follicle via CXCL13 secreted by the FDCs [1,38]. b. S1PR1 expression increases as the naïve B-cell matures, as long as it is not activated by an antigen. This allows the B-cell to move out of the lymph node and towards new lymphoid tissue [39,40]. 3. Activate upon exposure to free-floating antigen or FDC-bound antigen when CD-21 expression is high enough. CD-21 is a measure of B-cell activaiton [41]. B-cell activation is primarily decreased by the anti-inflammatory properties of IL-10.
Activated B-Cells	1. Created when a naïve B-cell is activated by a free (unbound) or FDC-bound antigen [1]. 2. Once activated, the B-cell will downregulate S1PR1 expression to prevent it from leaving the follicle [40]. 3. Activated B-cells can progress through one of two pathways: T-independent (Ti) response and T-dependent (Td) response [1]. 4. The forking point in Ti and Td pathways is determined by the type of antigen encountered. Ti antigen such as protein antigen will induce the Ti pathway. Td antigen such as LPS and repetitive epitopes will induce the Td pathway [1]. 5. Activated B-cells undergoing the T-dependent response will upregulate CCR7 and EBI2R, which use CCL19 and EBI2 Ligand as ligands, respectively. These two chemokines are produced by cells in the lymph node paracortex, and allow the activated B-cell to localize to the paracortex to interact with a helper T-cell (Tfh or Th2) [1]. a. If the activated B-cell finds a helper T-cell, it will go on to become a germinal center cell [1,42]. Activated B-cells undergoing the T-dependent response will become germinal center B-cells. 6. The T-independent response cannot become a germinal center response. Instead, the T-independent activated B-cell will differentiate into short-lived plasma cells [43].
Germinal Center B-Cells	1. These are differentiated from activated B-cells undergoing a T-dependent response, and represent the cells undergoing a germinal center response [1]. 2. The germinal center B-cell is able to produce both long-lived plasma cells and memory B-cells [44–46]. 3. The lifespan of each germinal center B-cell is set at 700 time steps (roughly 14 days) in order to calibrate the net total length of the germinal center response to be 3 weeks in duration [47].
Short-Lived Plasma Cells (SLPCs)	1. SLPCs have a total lifespan of 240 time steps, or 5 days [48]. 2. Secrete antibodies at a constant rate [1, 42].
Long-Lived Plasma Cells (LLPCs)	1. LLPCs have a total lifespan of 8000 time steps, or roughly 6 months [48]. 2. Secrete antibodies at a constant rate [1, 42].
Memory B-Cells	1. Circulate through the lymph node follicles using CXCR5 and S1PR1 to dictate entry and egress, similar to the naïve B-cell [38, 39]. 2. Activate upon antigen exposure via free-floating antigen or FDC-bound antigen when CD-21 expression is high enough. CD-21 is a measure of B-cell activation [41]. B-cell activation is primarily decreased by the anti-inflammatory properties of IL-10.
Regulatory B-Cells	1. Can be differentiated from naïve B-cells, memory B-cells, short-lived plasma cells, and long-lived plasma cells. Differentiation is induced by IL-6 and IL-21 [34]. 2. Secretes IL-10 [8].
Follicular T-Helper (Tfh) Cells	1. Patrols the follicle-paracortex border using its receptors CXCR5, CCR7, and EBI2R [49]. 2. Secretes IL-2, IL-4, IL-10, and IL-21 [50–52]. 3. If it comes into contact with an activated B-cell undergoing a T-dependent response, it allows the B-cell to form a germinal center B-cell [1, 49].
T-Helper 2 (Th2) Cells	1. Patrols solely the paracortex space using its receptors CCR7 and EBIR2 [1]. 2. Secretes IL-4 [53] and IL-10 [51]. 3. If it comes into contact with an activated B-cell undergoing a T-dependent response, it allows the B-cell to form a germinal center B-cell [1].
T-Helper 1 (Th1) Cells	1. Patrols solely the paracortex using its receptors CCR7 and EBIR2 [1]. 2. Secretes IFN-γ [51].
Antigen	1. A manipulatable number of antigen are inoculated into the the afferent lymphatic region of the follicle [1]. 2. At the same time, a proportional amount of IL-6 and TNF-α are added to the system as markers/mediators of sepsis. A large amount of antigen results in large amounts of IL-6 and TNF-α introduced, which corresponds with sepsis [54].
Antibody	1. Produced by SLPCs and LLPCs [1]. 2. Each antibody has a lifespan of 900 time steps, or roughly 20 days [55]. 3. Antibodies currently have no downstream function, but rather, are used as a metric of level of immunity generation.

### Overview of ABM Architecture

**Fig 1:**
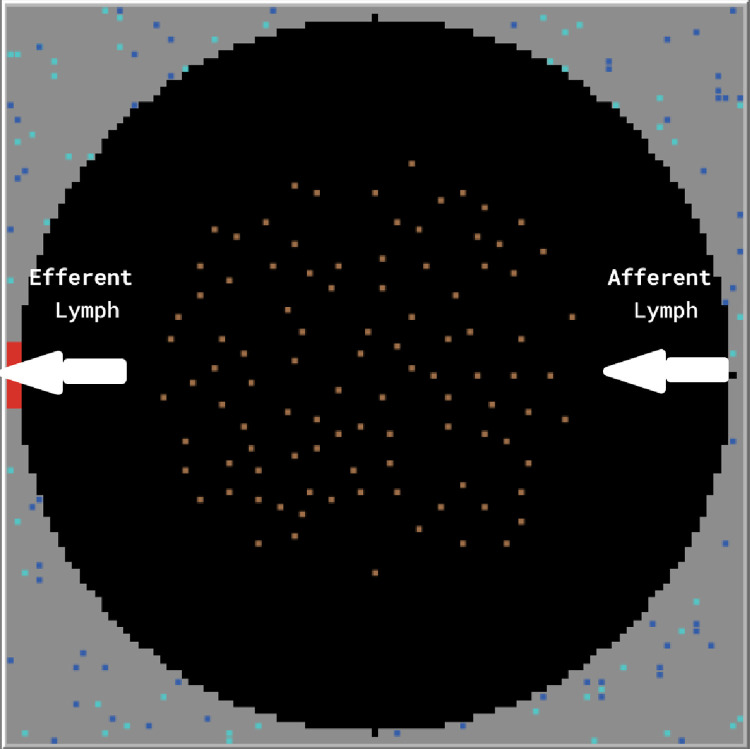
NetLogo grid-space layout. The black area represents the lymph node follicle, and the gray area represents the surrounding paracortex tissue. Each brown dot represents a follicular dendritic cell. Each blue and teal circle represents a helper T-cell. The afferent lymph flows into the follicle from the right side of the follicle and efferent lymph flows out on the left.

The BCIABM represents a two-dimensional model of a single lymph node follicle and its surrounding paracortex. It is constructed as a 100x100 2-dimensional square grid, where each grid space represents a space within the follicle that can contain antigen, inflammatory cells, and cytokines. The follicle includes one afferent and one efferent lymph vessel, which carry lymph containing cytokines and antigen to and from the follicle (Fig 1). In the model, one timestep represents thirty minutes in the real world. The model is not intended to have quantitative spatial fidelity to a real lymph node, but rather to depict an interaction space where some of the stochasticity present in cell-cell interactions can be represented. Therefore, the grid size is arbitrary and the time step is used to specify/define the molecular events. The movement across the space of the model is essentially calibrated to the needed cellular interactions for the required system-level outputs resulting from the simulated molecular/cellular events. As such, there is no calibration data to the actual movement rates of cells within the lymph node, the absolute values are not necessary, and it is only the relative relationships of these parameters relevant. This is analogous to the relational structure in ODE models between experimentally identifiable parameters and the invariably larger number of unidentifiable parameters that nonetheless are present and required in the model.

The BCIABM was implemented using NetLogo 6.3 [32], which can be downloaded through the following link: https://ccl.northwestern.edu/netlogo/.

### Cytokine/mediator implementation

In NetLogo, each grid space (called a patch) contains “patch variables” which are essentially state variables assigned to that patch. Patch variables were used to implement cytokine levels of Interleukin-2 (IL-2), Interleukin-4 (IL-4), IL-6, IL-10, Interleukin-21 (IL-21), Interferon-gamma (IFN-γ), and TNF-α in each grid space. These cytokines are secreted by the various cells that are present in the model based on the rules described in Tables 1 and 2. Once in the extracellular environment, diffusion of these mediators is implemented via NetLogo’s default “diffuse” function, which takes a defined percentage of the mediator variable from a patch and then evenly distributes that amount to the each of the surrounding eight patches (Moore Neighborhood). The degradation rate of these molecules is defined as fixed percentages during each time step. The cytokines then act on other cells to induce actions such as differentiation, apoptosis, and anergy.

**Table 2:** Cytokines, Chemokines, and their Functions.

Mediator	Source	Function
IL-2	Tfh Cells [52]	No downstream function currently, but included in the model for future work.
IL-4	Tfh Cells [50] and Th2 Cells [53]	Induces B-cell differentiation into memory B-cells [56], and Th0 cell differentiation into Th2 cells [57].
IL-6	FDCs [37] and systemic inflammation [9, 10, 58]	Affects naïve and memory B-cell CD21 activation level [5] and induces differentiation into regulatory B-cell [34].
IL-10	Regulatory B-Cells [8]	Inhibits naïve and memory B-cell activation [8].
IL-21	Tfh cells [50]	Induces differentiation into regulatory B-cells [34].
IFN-γ	Th1 cells [51]	Induces differentiation into plasma cells [59].
TNF-α	Activated B-cells [60] and systemic inflammation [9,10,58]	Induces B-cell apoptosis [7].
S1P1	Static gradient that is highest in concentration in the follicle exit [1]	Induces naïve B-cell exit from the follicle after a time limit [1].
CXCL13	Follicular dendritic cells [1]	Attracts B-cells towards the follicular dendritic cells [1].
CCL19	Stromal cells in paracortex [61]	Attracts activated B-cells towards the follicle-paracortex border for T-dependent responses [1].
EBI2	Stromal cells in paracortex [1]	Attracts activated B-cells towards the follicle-paracortex border for T-dependent responses [1].

### Antigen and antibody implementation

Antigens were implemented as agents, similar to B- and T-cells, that move throughout the follicle with random directionality. Although antigens do not have any associated state variables, they were implemented as agents in order to model and investigate the stochastic and spatial nature of antigen neutralization caused by direct collisions between B-cells and antigen.

Similarly, antibodies were implemented as agents as well, although the current model does not include functions like antigen neutralization or opsonization that require collision calculation. Instead, antibodies were implemented as agents in order to monitor each antibody’s total and remaining lifespan after it is produced by a plasma cell. This allows us to model the antibody titer decay as a direct function of agent death, rather than as an approximated mathematical decay curve. More importantly, though, it facilitates the addition of antibody functionality of antigen neutralization in future iterations of the model.

### Calibration

The BCIABM was calibrated in two sets of simulations. The first set of simulations aimed to calibrate accurate responses to mild antigen stimuli that are easily neutralizable. The second set of simulations aimed to calibrate B-cell responses to a severe antigen challenge representing the infectious load seen during sepsis.

In each of the mild and severe antigen challenge simulations, there were two periods of antigen introduction: the first on day 10 (time step 480) and the second on day 60 (time step 2880). The first inoculation of antigen was performed to set up a resting population of B-cell lineages, since day 0 represents a B-cell immune system that has never been exposed to any antigen and only consists of naïve B-cells. This first inoculation is analogous to a “wild-type”, sensitizing first exposure to a novel antigen. Then, once the baseline population cells was established, a second antigen stimulus was given on day 60. The magnitude of this second stimulation was how we distinguished between the mild and septic simulations. For the mild antigen stimulus, we used 50 antigen (arbitrary units), which represents an abstracted level of recoverable antigen exposure. For the sepsis simulation, we introduced a four-fold increase in antigen number of 200 (arbitrary units) to represent the severe antigen challenge seen during sepsis.

The expected behavior for the mild antigen exposure was for the B-cell response on the second antigen stimulus to be larger in magnitude than the first stimulus administered to establish a resting B-cell population. This would represent sensitization to an antigen following naïve exposure, reflected as larger responses in all B-cell subtypes: SLPCs, LLPCs, memory B-cells, and regulatory B-cells [44].

Next, the BCIABM was calibrated to severe antigen challenges seen during sepsis. The expected behavior would be a significant level of B-cell apoptosis induced by the influx of TNF-α, reflecting the systemic pro-inflammatory response seen in the initial phases of sepsis [6]. Both memory and naïve B-cells would downregulate their expression of CD21, which corresponds to a decrease in their activation level and thus produces the B-cell anergy seen during sepsis. Finally, there would be an increased rate of differentiation into regulatory B-cell and a consequent increase in IL-10 production [5], which induces the prominent anti-inflammatory signal to the septic stimulus. B-cell apoptosis, anergy, and differentiation into regulatory B-cells contribute to a prolonged period of B-cell immunosuppresion where we see very little B-cell activity. We expected to see immunosuppresion that lasted roughly 30 days [6] before enough antigen is neutralized by other means (in this case, follicular dendritic cell phagocytosis) such that the B-cells regain enough activity to neutralize.

### Simulation of Ongoing Background Inflammation (Chronic Disease)

Once the model was calibrated to reflect accurate physiologic responses to both mild and septic levels of antigen stimuli, we ran a set of simulation experiments to investigate B-cell behaviors during states of background inflammation. Low-level background inflammation consistent with aging and chronic disease was implemented as increasing steady-state levels of IL-6 and TNF-α, which are the dominant inflammatory cytokines present during sepsis and chronic inflammatory conditions like aging, diabetes, and obesity [62,63]. We were particularly interested in the downstream effects of increasing IL-6 and TNF-α levels as they are known to negatively correlate with functional ability and immune responsiveness in elderly patients and patients with chronic disease [13]. In these simulations, both the first and second exposures were set as exposures of 50 antigen (aribtrary units) in order to simulate mild antigen challenges that are easily neutralized in healthy patients. We measured the efficacy of the B-cell response to the first and second antigen exposures separately by using the maximumum cell counts of each B-cell subtype created by each exposure.

### Results

The BCIABM successfully integrates and models the B-cell response to both mild and severe antigen challenges with respect to the following B-cell subtypes: short-lived plasma cells, long-lived plasma cells, memory B-cells, and regulatory B-cells. In addition, it successfully implements the cytokine influences of IL-2, IL-4, IL-6, IL-10, IL-21, TNF-α, and IFN-γ.

### Calibration of the Control: 1^st^ Sensitizing Exposure, 2^nd^ Mild Exposure

**Fig 2:**
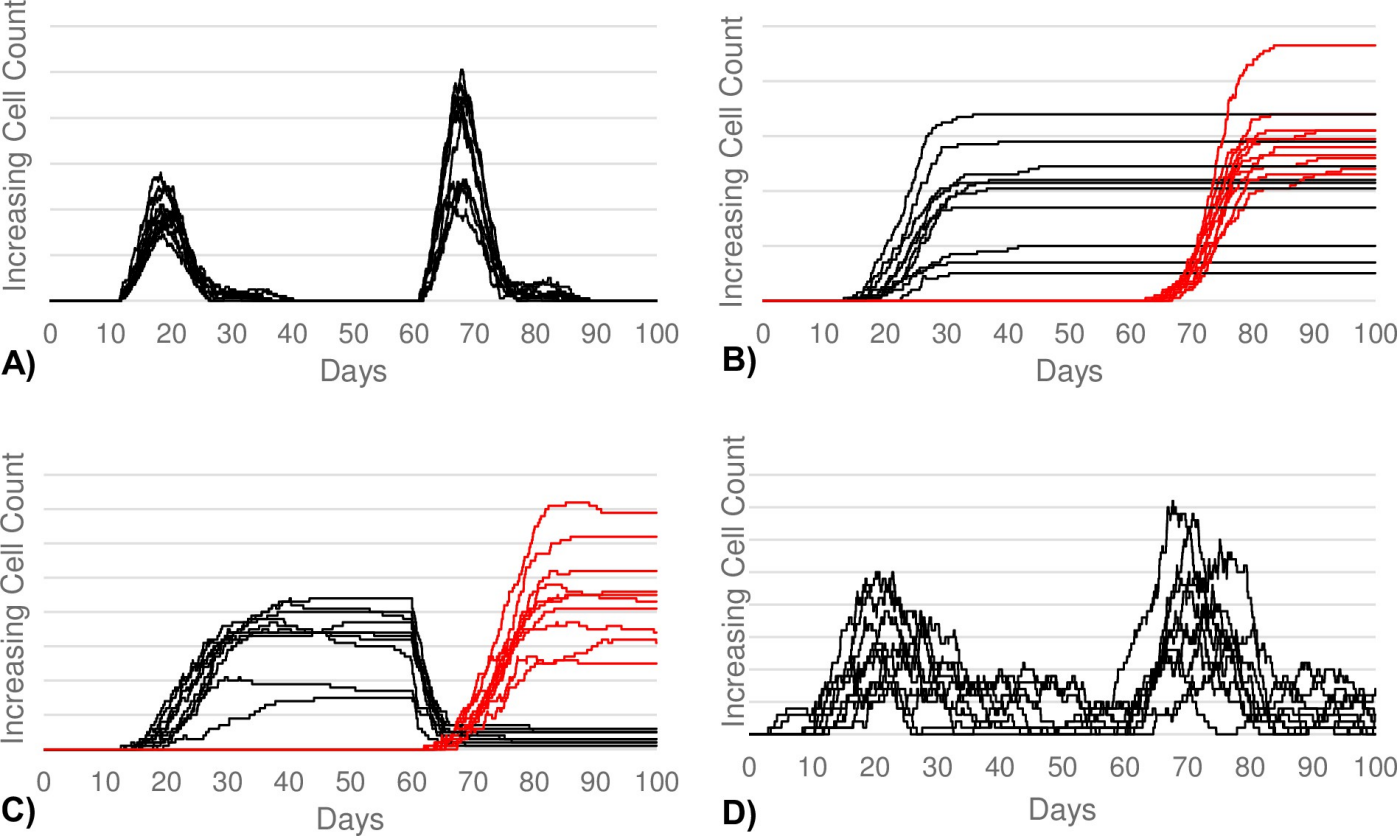
B-cell responses to mild antigen stimuli. (A) Demonstrates the SLPC response. (B) Demonstrates the LLPC response. (C) Demonstrates the memory B-cell response. (D) Demonstrates the regulatory B-cell response. All four B-cell subtypes show a larger response upon second antigen exposure (day 60) compared to the first exposure (day 10). (B, C) The cells produced after the first and second exposure were color-coded black and red, respectively, in order to distinguish the magnitude of response for each exposure. This is necessary since the LLPCs and memory B-cells produced after the first exposure persist well into the second exposure. (A, D) The majority, if not all, of the cells produced after the first exposure die prior to the second exposure.

The BCIABM was first calibrated to reflect accurate responses to mild antigen stimuli. The goal was to see a larger immune response upon 2^nd^ antigen exposure in all four of the B-cell subtypes of SLPCs, LLPCs, memory B-cells, and regulatory B-cells. This represents immune sensitization to a previously seen antigen. These calibration goals were met (see Fig 2).

**Fig 3:**
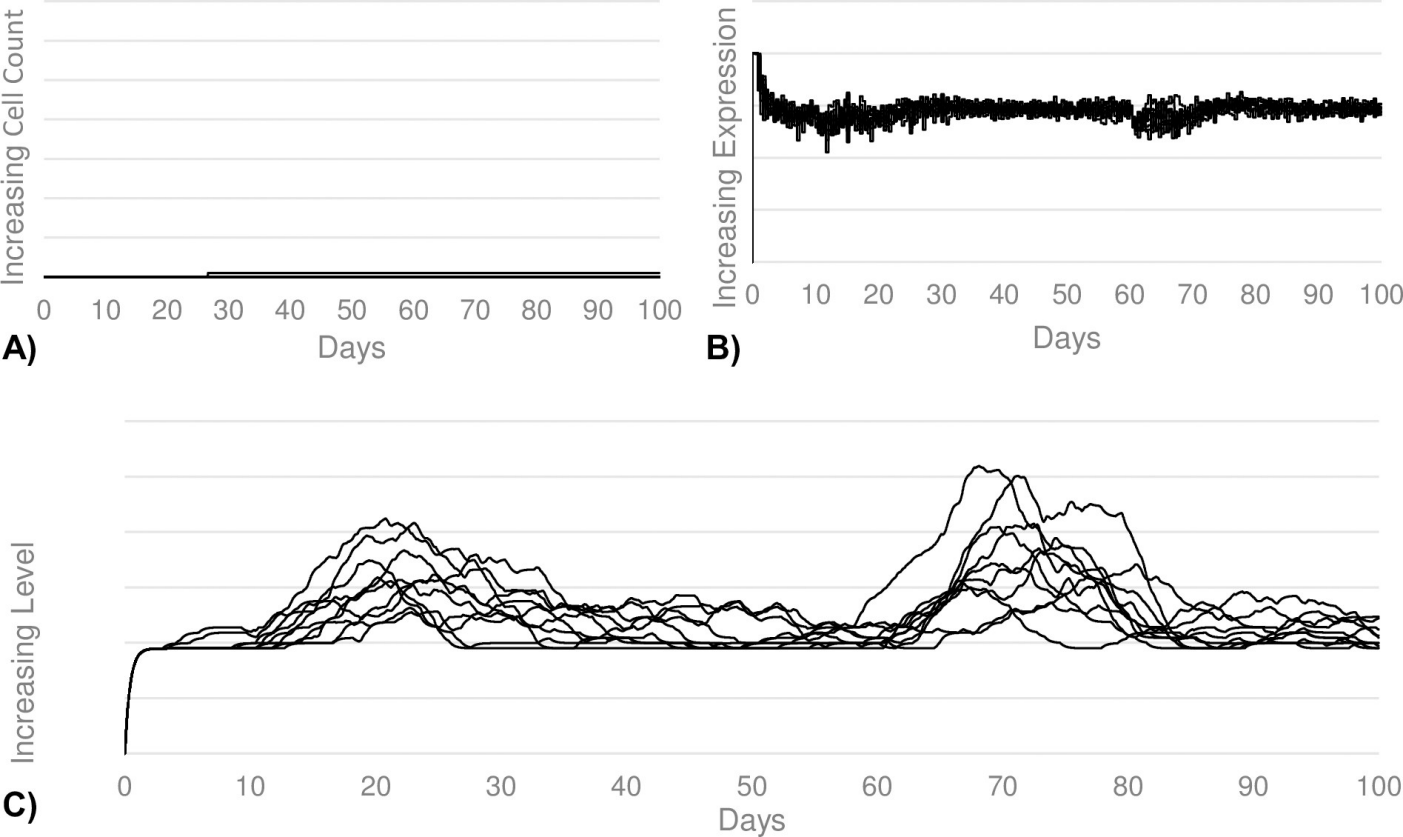
B-cell apoptosis, activation, and IL-10 levels during mild antigen stimuli. (A) Shows the total number of B-cells that undergo apoptosis. There is no appreciable level of apoptosis upon first or second exposures. (B) Shows the average CD21 expression by naïve and memory B-cells. There is no change in CD21 expression upon first or second exposures. (C) Shows the total level of IL-10 secreted by regulatory B-cells. There is a slight increase in the IL-10 levels following second exposure compared to the first exposure, corresponding to the increased regulatory B-cell differentiation.

In addition to the cell population counts, we also measured IL-10 secretion by regulatory B-cells, CD21 expression by B-cells, and level of apoptosis. These parameters are more relevant to the sepsis calibrations, but we first needed a control simulation for comparison. Here, our calibration goal was to see a mild increase in IL-10 secretion corresponding to the mild increase in regulatory B-cells. Additionally, we wanted to see little to no decrease in the CD21 expression of B-cells since there is no septic state to induce B-cell anergy. Finally, we wanted to see no signficant level of B-cell apoptosis since there is not elevated TNF-α present in the system. These calibration goals were met (see Fig 3).

### Calibration of Sepsis: 1^st^ Sensitizing Exposure, 2^nd^ Severe Exposure

**Fig 4:**
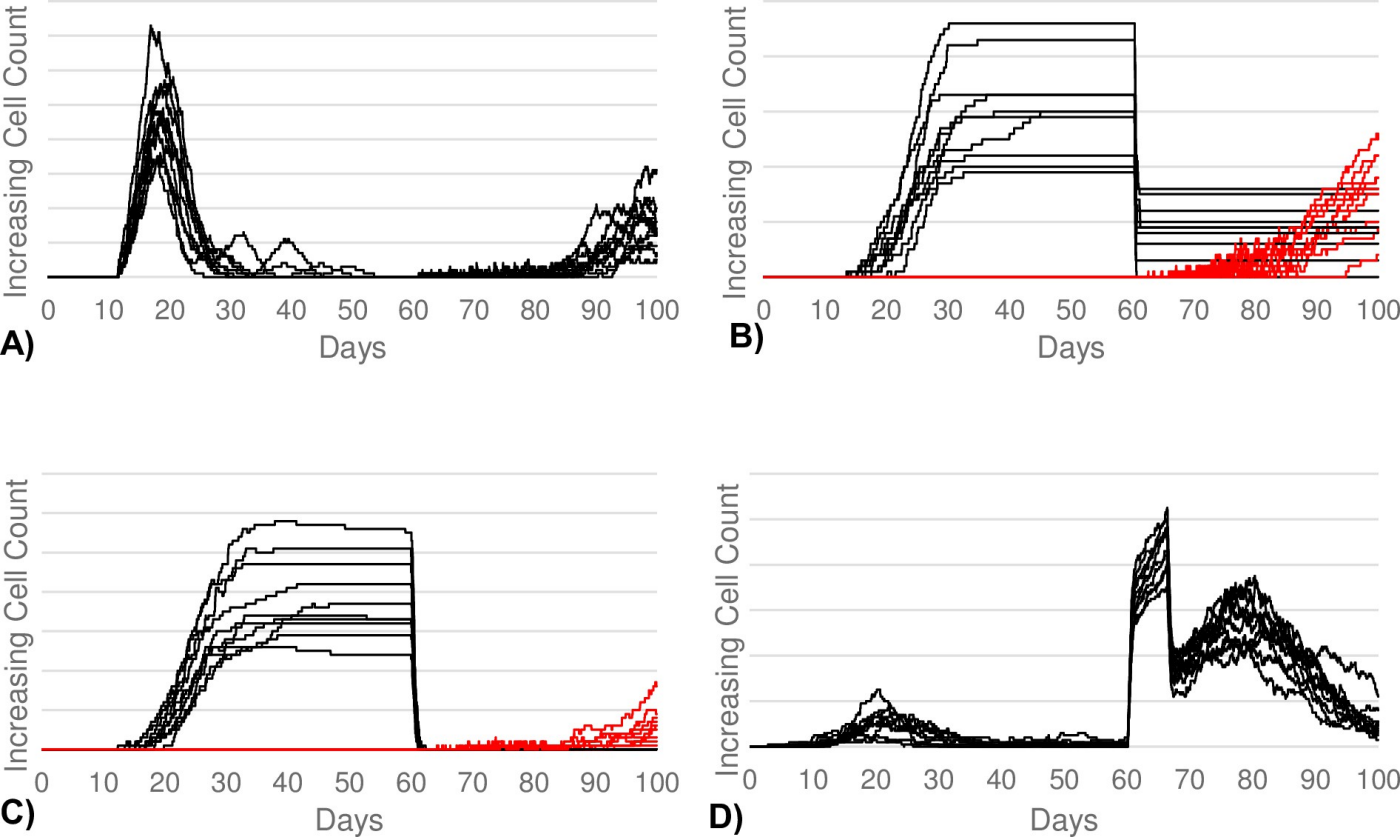
B-cell responses to severe antigen challenges seen in sepsis. (A) Demonstrates the SLPC response. (B) Demonstrates the LLPC response. (C) Demonstrates the memory B-cell response. (D) Demonstrates the regulatory B-cell response. The SLPCs, LLPCs, and memory B-cells demonstrate a roughly 30-day period of low activity after the second exposure on day 60 corresponding to immunosuppresion. On the contrary, the anti-inflammatory regulatory B-cells demonstrate a large spike in activity. (B, C) The cells produced after the first and second exposure were color-coded black and red, respectively, in order to distinguish the magnitude of response for each exposure. This is required since the LLPCs and memory B-cells produced after the first exposure persist well into the second exposure. (A, D) The majority, if not all, of the cells produced after the first exposure die prior to the second exposure.

In the simulations of sepsis, the 2^nd^ exposure was modified to be a larger, more severe antigen challenge. The 1^st^ exposure was kept constant from the control simulations in order to create similar post-first-exposure B-cell population counts, while the 2^nd^ exposure dose was increased as the independent variable. Our primary calibration goal was to see a prolonged period of low B-cell activity in the SLPC, LLPC, and memory B-cell subtypes for roughly 20 to 30 days after the second exposure. The model behavior showed that the SLPC and memory B-cell levels did not begin to significantly increase until roughly 25 days post-exposure. Similarly, the LLPC population did not begin to significantly increase until roughly 20 days post-exposure. On the other hand, we wanted to see an immediate and large increase in the differentiation into regulatory B-cells, which represents the large anti-inflammatory response to the septic insult. Here, we see a nearly five-fold increase in the regulatory B-cell population compared to the first exposure response (see Fig 4). In contrast, there was only an approximate 1.5x increase during the control simulations.

**Fig 5:**
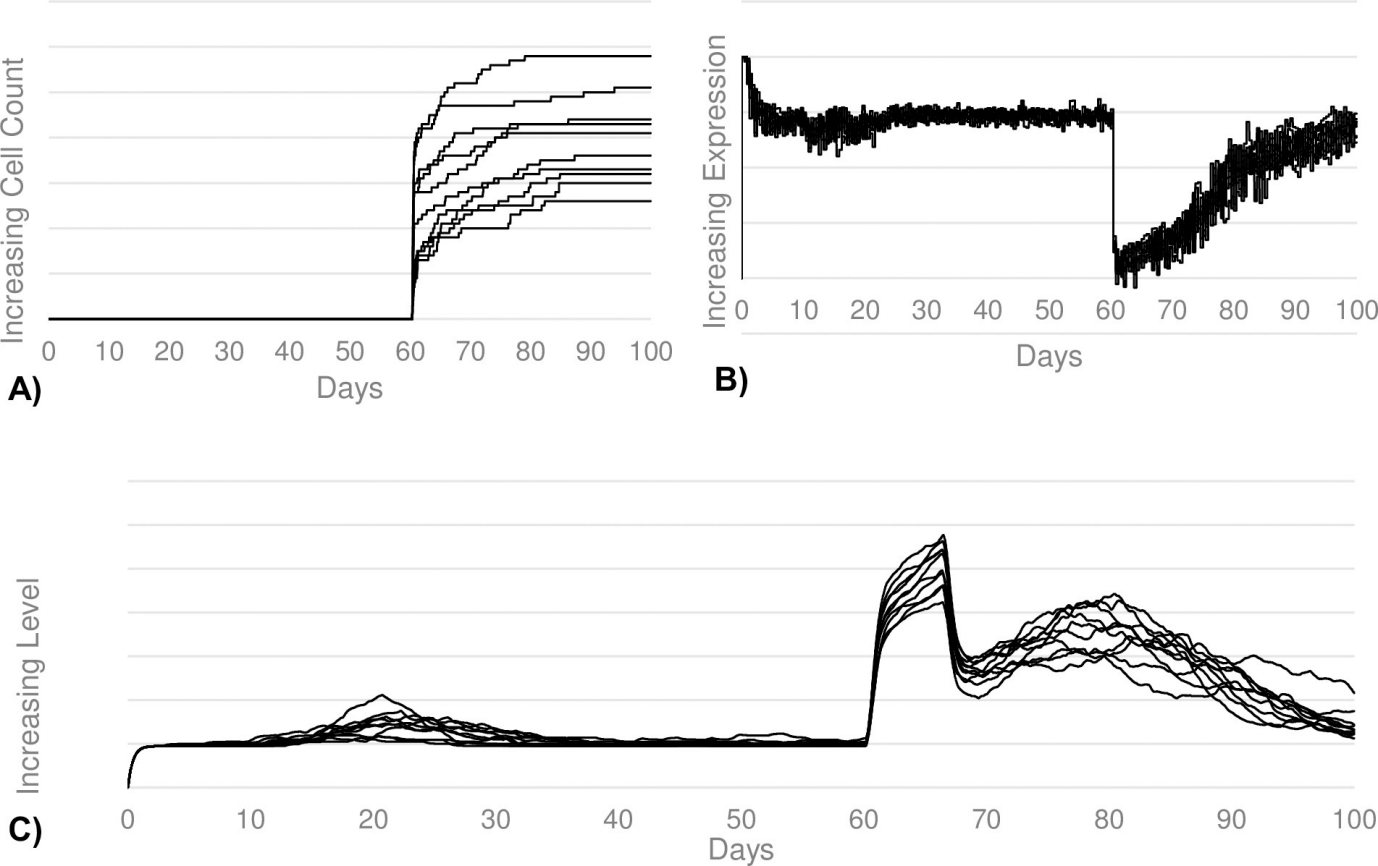
B-cell apoptosis, activation, and IL-10 during sepsis. (A) Shows the total number of B-cells that undergo apoptosis. There is a significant increase in apoptosis after the septic antigen challenge on day 60. (B) Shows the average CD21 expression by naïve and memory B-cells. There is a significant decrease in CD21 expression, and therefore, level of activation, upon the septic antigen challenge. (C) Shows the total level of IL-10 secreted by regulatory B-cells. There is a large increase in the IL-10 levels following the septic exposure.

Additionally, our calibration goals for the sepsis simulations included IL-10 secretion, CD21 expression levels, and apoptosis. Our goals were to see a significantly increased IL-10 secretion corresponding to the larger regulatory B-cell population, significantly diminished CD21 expression representing the B-cell anergy induced by IL-6 seen during sepsis, as well as a large rise in the total level of B-cell apoptosis induced by TNF-α seen during sepsis. Our calibration goals in all three of these categories were met (see Fig 5). We see a nearly 5-fold increase in IL-10 secretion upon sepsis induction, corresponding to the 5-fold increase in regulatory B-cell count. We also see a significant decrease in CD-21 expresion, which does not recuperate to normal levels until roughly 30 days after sepsis exposure. Finally, there is a sharp increase in the total level of apoptosis.

### Validation Experiments with Background Inflammation (Chronic Disease)

**Fig 6:**
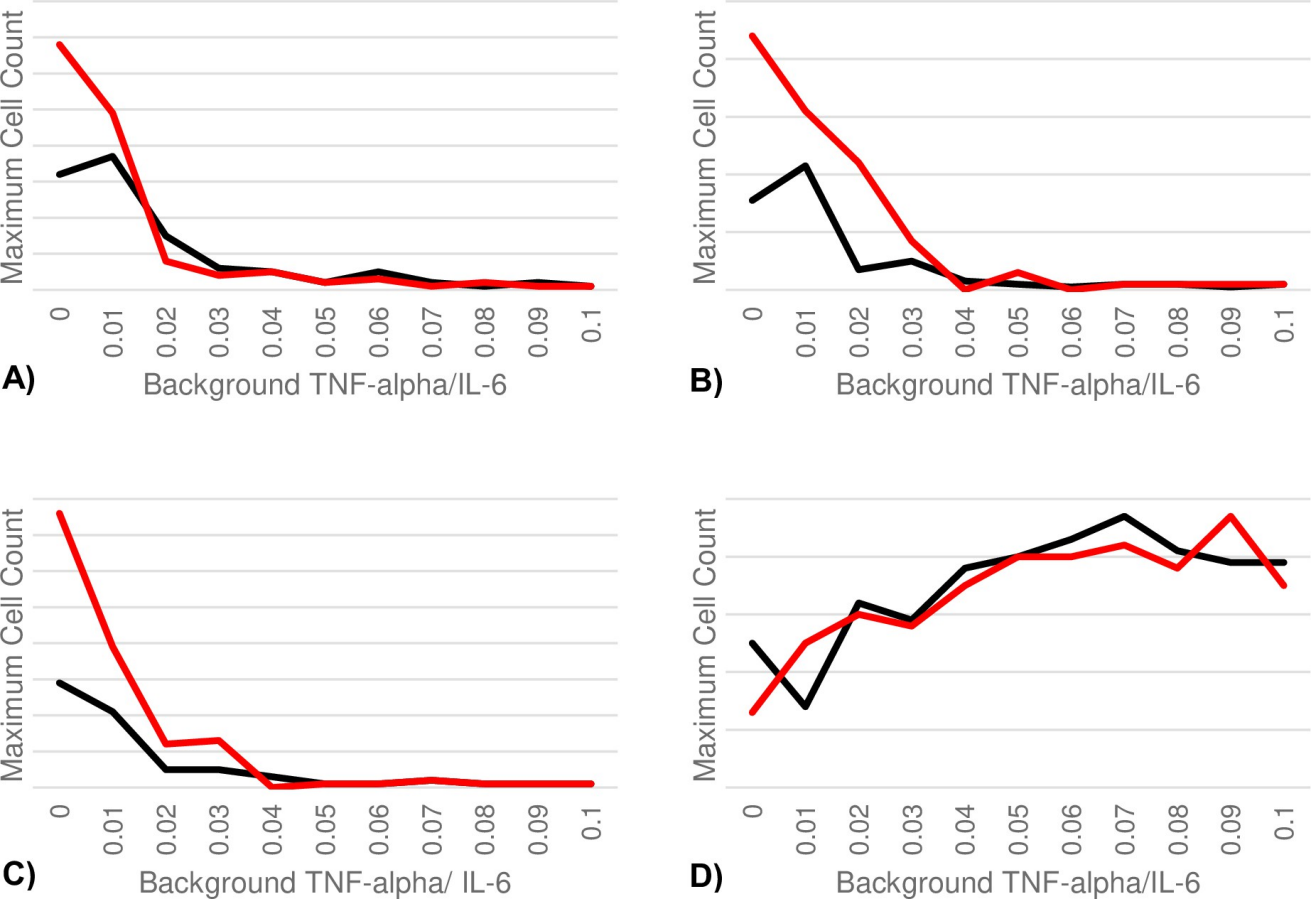
B-cell responses amidst increasing levels of background inflammation. In all panels, the black line represents the B-cell response to the first exposure while the red line represents the response to the second exposure. (A) Shows the maximum SLPC responses in relation to background inflammation. (B)Shows the maximum LLPC responses. (C) Shows the maximum memory B-cell responses. (D) Shows the maximum regulatory B-cell responses.

In the B-cell responses to both the first and second antigen exposures, we see a decrease in maximum B-cell response in SLPCs, LLPCs, and memory B-cell populations as the overall level of background TNF-α and IL-6 increase. This represents a decreased efficacy of the antigen-neutralizing B-cell response with increasing levels of background inflammation. On the other hand, we see a steady increase in the maximum regulatory B-cell count with increasing background inflammation, induced by a similar IL-6-mediated mechanism that caused the elevated regulatory B-cell counts seen during the sepsis simulations. These findings match current literature showing regulatory B-cell induction during disease and autoimmunity [64]. The steadily elevated IL-6 and TNF-α levels induce a chronic state of B-cell anergy and apoptosis mediated by regulatory B-cells (see Fig 6).

## Discussion

The B-cell immune system is composed of a highly intricate network of cells, cytokines and chemokines, and antigen that interact with one another to neutralize antigen and confer future immunity. Due to the complexity of the system, it can be diffult to fully appreciate the nuances of each interaction, especially when the influence of ongoing inflammation is considered. Fortunately, agent-based models presents a unique solution that integrates all of the complex steps in immunity generation. The BCIABM succesfuly models the B-cell response during both mild antigen stimuli and severe antigen challenges in regards to the behaviors of SLPCs, LLPCs, regulatory B-cells, and memory B-cells. In addition, it integrates the various cytokine influences that determine B-cell differentiation.

When we simulated the B-cell response amidst chronically increased baseline inflammation, we found diminishing B-cell efficacy, which in real-world patients would manifest as poor antibody and memory formation. In fact, the results may represent an underlying mechanism for the findings that aging populations or patients with diabetes or obesity have a greater susceptibility to re-infection with viruses like SARS-CoV-2 due to impaired antibody and memory formation. In these populations, the chronically elevated baseline inflammation is primarily mediated by IL-6 and TNF-α [62,63], which is known to induce a state of impaired immune responsiveness in B-cells [13] and thus results in the phenotype of frequent primary infections and re-infections. Specifically, we draw attention to a study [65] in which the authors demonstrated increasing systemic sequelae effects from repeated SARS-CoV-2 infections in a population with increased levels of background inflammation compared to the general population with respect to obesity-status, current/former smoker status, and disease state (e.g., chronic kidney disease, cardiovascular disease, diabetes). Importantly, the results of the above-referenced study were not considered in the calibration or development of the BCIABM, which further validates the mechanisms and abstractions present in this model.

A similar phenomenon is seen when testing efficacy of vaccines for patients in developing countries, who due to lack of quality care, are more likely to have underlying inflammatory diseases that impair the B-cell response to endemic infections. This can unfortunately decrease the efficacy of vaccines developed in wealthier, more developed countries once they are transferred to these developing countries.

Overall, this model gives rise to a platform that can easily be manipulated to closely study B-cell behaviors with greater granularity since agent-based modeling specifically allows examination of the characteristics of each individual cell, or agent, and its interactions with other components of the system. Future work for the project includes further calibration of the model using additional real-world data in order to refine how closely the model reflects real B-cell behavior. Additionally, we aim to explore how vaccine efficacy is affected by states of background inflammation. Ultimately, we hope to utilize this model as a tool to predict the B-cell response to an array of vaccines in order to enhance development of efficacious vaccines against infectious diseases.

## Supporting information

S1 Text Parameter Sensitivity Analysis. (DOCX)

S1 Table Parameter Ranges and Intervals. This table shows the ranges of parameters that were swept over to calibrate the model.
(DOCX)

S2 Table Effects of Manipulating Parameters on the Mild Antigen Stimulus Simulations. This table provides detailed descriptions of the effects of the parameters in S1 Table when the model is exposed to a mild antigen load.
(DOCX)

S3 Table Effects of Manipulating Parameters on the Severe Antigen Challenge Simulations. This table provides detailed descriptions of the effects of the parameters in S1 Table when the model is exposed to a significant/severe antigen load.
(DOCX)

S1 Fig The effects of changing CD-21 activation thresholds by cell type. In all panels, the increasingly saturated blue lines represent cell responses to decreasing CD-21 activation thresholds over which naïve and memory B-cells activate. The red line in each panel represents the median value of incremented thresholds, which was set as the baseline threshold to which to compare. Panels **A**, **C**, **E**, and **G** demonstrate the responses to the mild antigen stimulus simulations for SLPCs, LLPCs, memory B-cells, and regulatory B-cells, respectively. Panels **B**, **D**, **F**, and **H** demonstrate the responses to the severe antigen challenge simulations for SLPCs, LLPCs, memory B-cells, and regulatory B-cells, respectively.
(TIF)

S2 Fig The effects of changing TNF-α-induced apoptosis thresholds by cell type. In all panels, the increasingly saturated blue lines represent cell responses to decreasing TNF-α thresholds over which cells undergo apoptosis. The red line in each panel represents the median value of incremented thresholds, which was set as the baseline threshold to which to compare. Panels **A**, **C**, **E**, and **G** demonstrate the responses to the mild antigen stimulus simulations for SLPCs, LLPCs, memory B-cells, and regulatory B-cells, respectively. Panels **B**, **D**, **F**, and **H** demonstrate the responses to the severe antigen challenge simulations for SLPCs, LLPCs, memory B-cells, and regulatory B-cells, respectively.
(TIF)

S3 Fig The effects of changing the IL-6 thresholds to differentiate into regulatory B-cells by cell type. In all panels, the increasingly saturated blue lines represent cell responses to decreasing IL-6 thresholds over which cells undergo differentiation into regulatory B-cells. The red line in each panel represents the median value of incremented thresholds, which was set as the baseline threshold to which to compare. Panels **A**, **C**, **E**, and **G** demonstrate the responses to the mild antigen stimulus simulations for SLPCs, LLPCs, memory B-cells, and regulatory B-cells, respectively. Panels **B**, **D**, **F**, and **H** demonstrate the responses to the severe antigen challenge simulations for SLPCs, LLPCs, memory B-cells, and regulatory B-cells, respectively.
(TIF)
