# Supplementary material for: Examining B-cell dynamics and responsiveness in different inflammatory milieus using an agent-based model
Source: PLoS Comput Biol. 2024 Jan 23;20(1):e1011776. doi: 10.1371/journal.pcbi.1011776 (PMC10805321; doi:10.1371/journal.pcbi.1011776)
Supplement: S1 Text — (DOCX) [file pcbi.1011776.s001.docx]

**S1 Text: Parameter Sensitivity Analysis**

The calibrations of the mild antigen stimulus simulations and severe, septic antigen challenge simulations were completed through a parameter sensitivity analysis which swept through an extensive set of parameter combinations in order to determine the optimal set of parameters that produces the desired B-cell behaviors. The parameters that were modified during the analysis included: the B-cell Cluster of Differentiation 21 (CD-21) expression threshold at which naïve and memory B-cells activate, the surrounding Tumor Necrosis Factor-alpha (TNF-α) threshold required to induce apoptosis in all B-cell lineages, and the surrounding Interleukin-6 (IL-6) threshold at which differentiation into regulatory B-cell occurs.

We used NetLogo’s Behavior Space tool to run 7040 unique simulations that incremented through every combination of the aforementioned parameters at constant intervals (Table S1). In each simulation, the short-lived plasma cell (SLPC), long-lived plasma cell (LLPC), memory B-cell, and regulatory B-cell counts at each point in time were recorded. The results were then parsed by utilizing a basic python script with the following steps. First, the script was used to find all parameter sets that satisfied our criteria for the mild antigen stimulus simulations – the maximum B-cell counts in all lineages should be larger in the second stimulus than in the first stimulus, representing antigen sensitization. There were roughly 2000 parameter sets that met this criterion. These 2000 sets were parsed via another python script to find those that fulfilled our criteria for the severe, septic antigen challenge simulations, namely that the parameters should produce a minimum of a 20-day period of SLPC, LLPC, and memory B-cell counts under ten immediately following the severe antigen challenge. The resulting 100 parameter sets represented the parameters that fulfilled the inclusion criteria for both the mild antigen stimuli and the severe antigen challenges. Finally, these 100 parameter sets were plotted with respect to each of the B-cell lineages and then manually visualized to qualitatively select the parameter set that produced the least irregularity in cell counts, unimodal shapes for the SLPC response, LLPC responses that were delayed with respect to the SLPC responses, and for the severe antigen challenge calibration specifically, a period of immunosuppresion as close as possible to 30 days in duration. Once the optimal parameter set was selected, we analyzed how the system responded as each parameter was manipulated away from the optimized value. The effects of modifying each parameter in the mild antigen stimuli and severe antigen challenge simulations are summarized in Table S2 and S3, respectively.

Overall, the parameter sensitivity allowed for a systematic selection of the most optimal values of the following parameters: the CD-21 activation threshold, the TNF-αinduced apoptosis threshold, and the IL-6-induced threshold of differentiation into regulatory B-cells. Once the optimal parameter set was found, it elucidated the changing behaviors of each B-cell subtype in response to increasing or decreasing the aforementioned thresholds.
