# Supplementary material for: Examining B-cell dynamics and responsiveness in different inflammatory milieus using an agent-based model
Source: PLoS Comput Biol. 2024 Jan 23;20(1):e1011776. doi: 10.1371/journal.pcbi.1011776 (PMC10805321; doi:10.1371/journal.pcbi.1011776)
Supplement: S1 Table — This table shows the ranges of parameters that were swept over to calibrate the model. (DOCX) [file pcbi.1011776.s002.docx]

**Table S1: Parameter Ranges and Intervals**

| Parameter | Minimum | Maximum | Interval |
| --- | --- | --- | --- |
| CD-21 Expression Activation Threshold | 50 | 150 | 10 |
| TNF-α Apoptosis Threshold | 240 | 310 | 10 |
| IL-6 Threshold for Differentiation into Regulatory B-Cells | 140 | 210 | 10 |
