# Supplementary material for: Examining B-cell dynamics and responsiveness in different inflammatory milieus using an agent-based model
Source: PLoS Comput Biol. 2024 Jan 23;20(1):e1011776. doi: 10.1371/journal.pcbi.1011776 (PMC10805321; doi:10.1371/journal.pcbi.1011776)
Supplement: S2 Table — This table provides detailed descriptions of the effects of the parameters in S1 Table when the model is exposed to a mild antigen load. (DOCX) [file pcbi.1011776.s003.docx]

**Table S2: Effects of Manipulating Parameters on the Mild Antigen Stimulus Simulations**

| Parameter | Effects of Increasing Threshold | Effects of Decreasing Threshold |
| --- | --- | --- |
| CD-21 Expression Activation Threshold | **Dampened responses** to first and second stimuli **in all B-cell subtypes**, causing decreased memory B-cell counts after the first stimulus and thus poorer sensitization to the antigen (Figure S1, Panel A, C, E, and G). | **Heightened responses** to first and second stimuli **in all B-cell subtypes** (Figure S1, Panel A, C, E, and G). |
| TNF-α Apoptosis Threshold | **No significant effects** on B-cell responses. This is likely due to the fact that TNF-α levels remain much lower than the thresholds included in the analysis, so apoptosis in these simulations was not an influencing factor (Figure S2, Panel A, C, E, and G). | **No significant effects** on B-cell responses. This is likely due to the fact that TNF-α levels remain much lower than the thresholds included in the analysis, so apoptosis in these simulations was not an influencing factor (Figure S2, Panel A, C, E, and G). |
| IL-6 Threshold for Differentiation into Regulatory B-Cells | **Increased B-cell responses in all subtypes except for the regulatory B-cells** (Figure S3, Panel A, C, E, G). This was due to the fact that when the threshold for differentiation was increased, fewer naïve B-cells, SLPCs, LLPCs, and memory B-cells differentiated into regulatory B-cells, resulting in decreased immunosuppressive effects. | **Decreased B-cell responses in all subtypes except for the regulatory B-cells** (Figure S3, Panel A, C, E, G). This was due to the fact that when the threshold for differentiation was lowered, more naïve B-cells, SLPCs, LLPCs, and memory B-cells differentiated into the regulatory B-cells that induced an immunosuppressive effect. |
