# Supplementary material for: Examining B-cell dynamics and responsiveness in different inflammatory milieus using an agent-based model
Source: PLoS Comput Biol. 2024 Jan 23;20(1):e1011776. doi: 10.1371/journal.pcbi.1011776 (PMC10805321; doi:10.1371/journal.pcbi.1011776)
Supplement: S3 Table — This table provides detailed descriptions of the effects of the parameters in S1 Table when the model is exposed to a significant/severe antigen load. (DOCX) [file pcbi.1011776.s004.docx]

**Table S3: Effects of Manipulating Parameters on the Severe Antigen Challenge Simulations**

| Parameter | Effects of Increasing Threshold | Effects of Decreasing Threshold |
| --- | --- | --- |
| CD-21 Expression Activation Threshold | **Decreased all B-cell responses** to both first and second stimuli and **slowed the rate of B-cell recovery** after the septic insult (Figure S1, Panel B, D, F, and H). | **Increased all B-cell responses** but **shortened durations of B-cell anergy** and immunosuppresion in the severe antigen challenge simulations, representing increased speed of B-cell recovery (Figure S1, Panel B, D, F, and H). |
| TNF-α Apoptosis Threshold | The **LLPC** response showed **decreased apoptosis** after the septic insult (Figure S2, Panel D). This behavior is not seen in any other cell type because the other cell types exist in a space that is much higher in overall TNF-α levels, so regardless of the threshold, the other cell types undergo apoptosis after the septic insult. | The **LLPC** response showed **increased apoptosis** after the septic insult (Figure S2, Panel D). This behavior is not seen in any other cell type because the other cell types exist in a space that is much higher in overall TNF-α levels, so regardless of the threshold, the other cell types undergo apoptosis after the septic insult. |
| IL-6 Threshold for Differentiation into Regulatory B-Cells | **Increased B-cell responses in all subtypes except for the regulatory B-cells** and **shortened** the period of immunosuppresion after the septic insult (Figure S3, Panel B, D, and F). | **Decreased B-cell responses in all subtypes except for the regulatory B-cells** and **prolonged** the period of immunosuppresion after the septic insult (Figure S3, Panel B, D, and F). |
