# Supplementary figures and images for: Examining B-cell dynamics and responsiveness in different inflammatory milieus using an agent-based model
Source: PLoS Comput Biol. 2024 Jan 23;20(1):e1011776. doi: 10.1371/journal.pcbi.1011776 (PMC10805321; doi:10.1371/journal.pcbi.1011776)

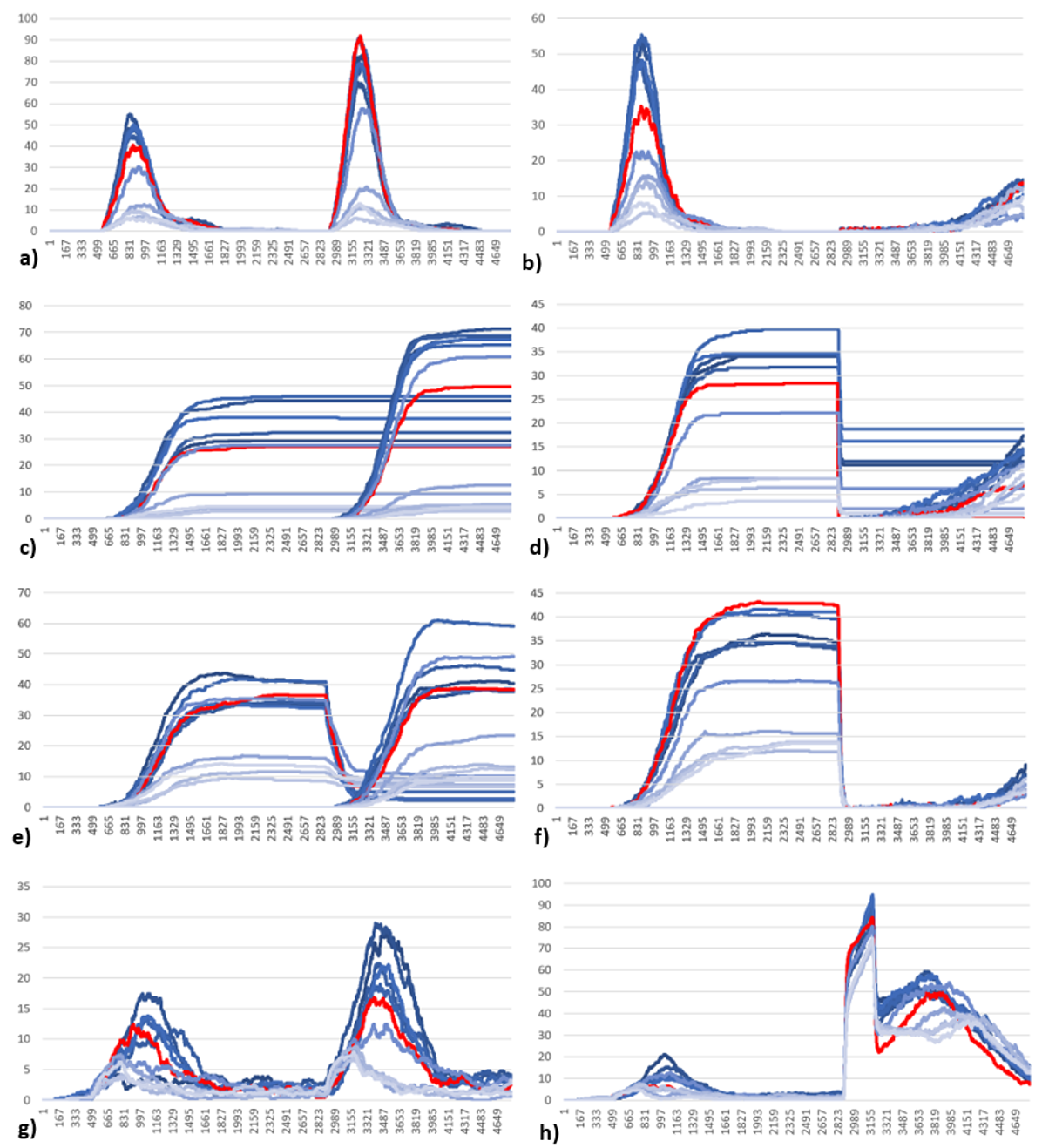

Supplement: S1 Fig — In all panels, the increasingly saturated blue lines represent cell responses to decreasing CD-21 activation thresholds over which naïve and memory B-cells activate. The red line in each panel represents the median value of incremented thresholds, which was set as the baseline threshold to which to compare. Panels A, C, E, and G demonstrate the responses to the mild antigen stimulus simulations for SLPCs, LLPCs, memory B-cells, and regulatory B-cells, respectively. Panels B, D, F, and H demonstrate the responses to the severe antigen challenge simulations for SLPCs, LLPCs, memory B-cells, and regulatory B-cells, respectively. (TIF) [file pcbi.1011776.s005.tif]

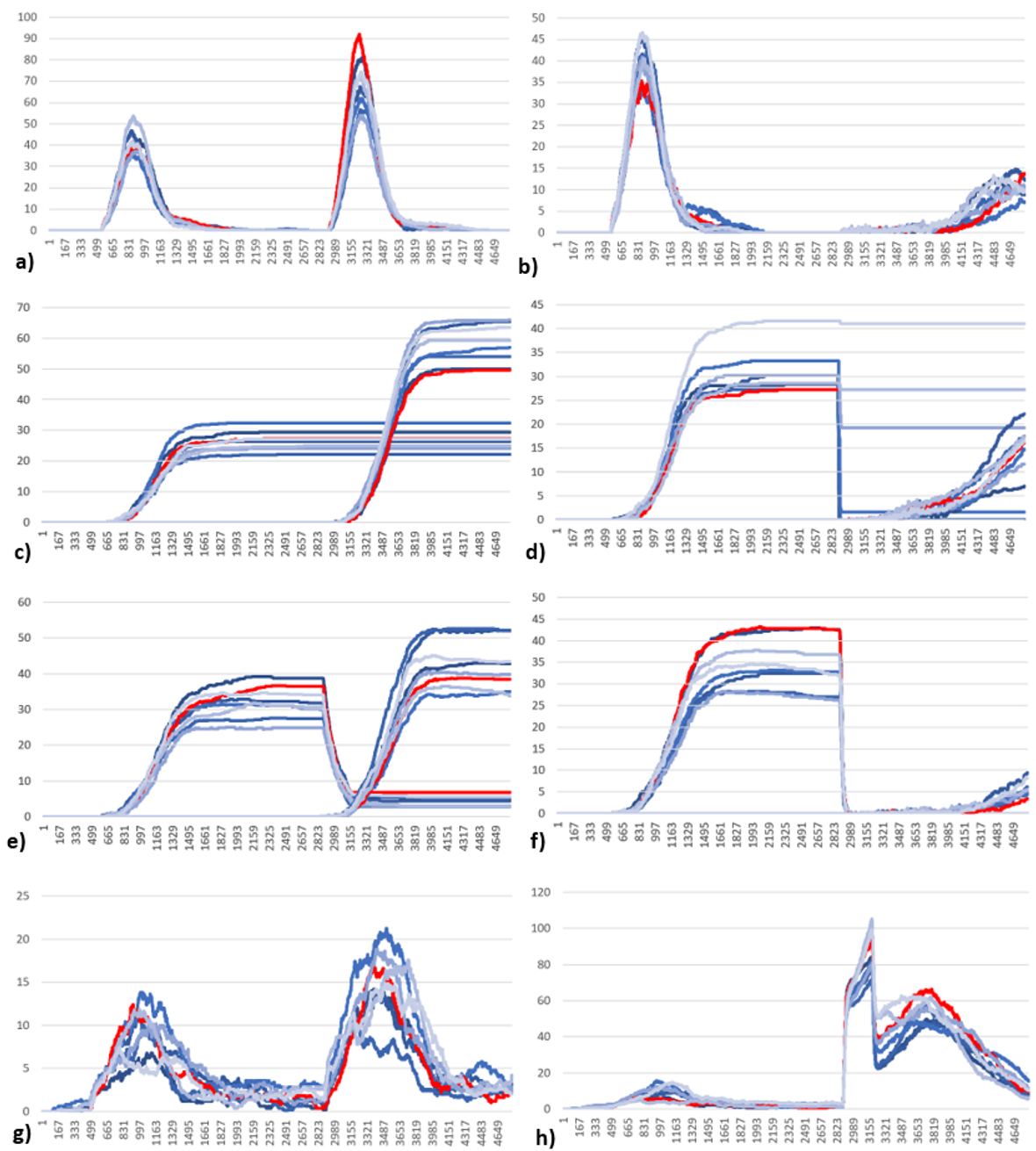

Supplement: S2 Fig — In all panels, the increasingly saturated blue lines represent cell responses to decreasing TNF-α thresholds over which cells undergo apoptosis. The red line in each panel represents the median value of incremented thresholds, which was set as the baseline threshold to which to compare. Panels A, C, E, and G demonstrate the responses to the mild antigen stimulus simulations for SLPCs, LLPCs, memory B-cells, and regulatory B-cells, respectively. Panels B, D, F, and H demonstrate the responses to the severe antigen challenge simulations for SLPCs, LLPCs, memory B-cells, and regulatory B-cells, respectively. (TIF) [file pcbi.1011776.s006.tif]

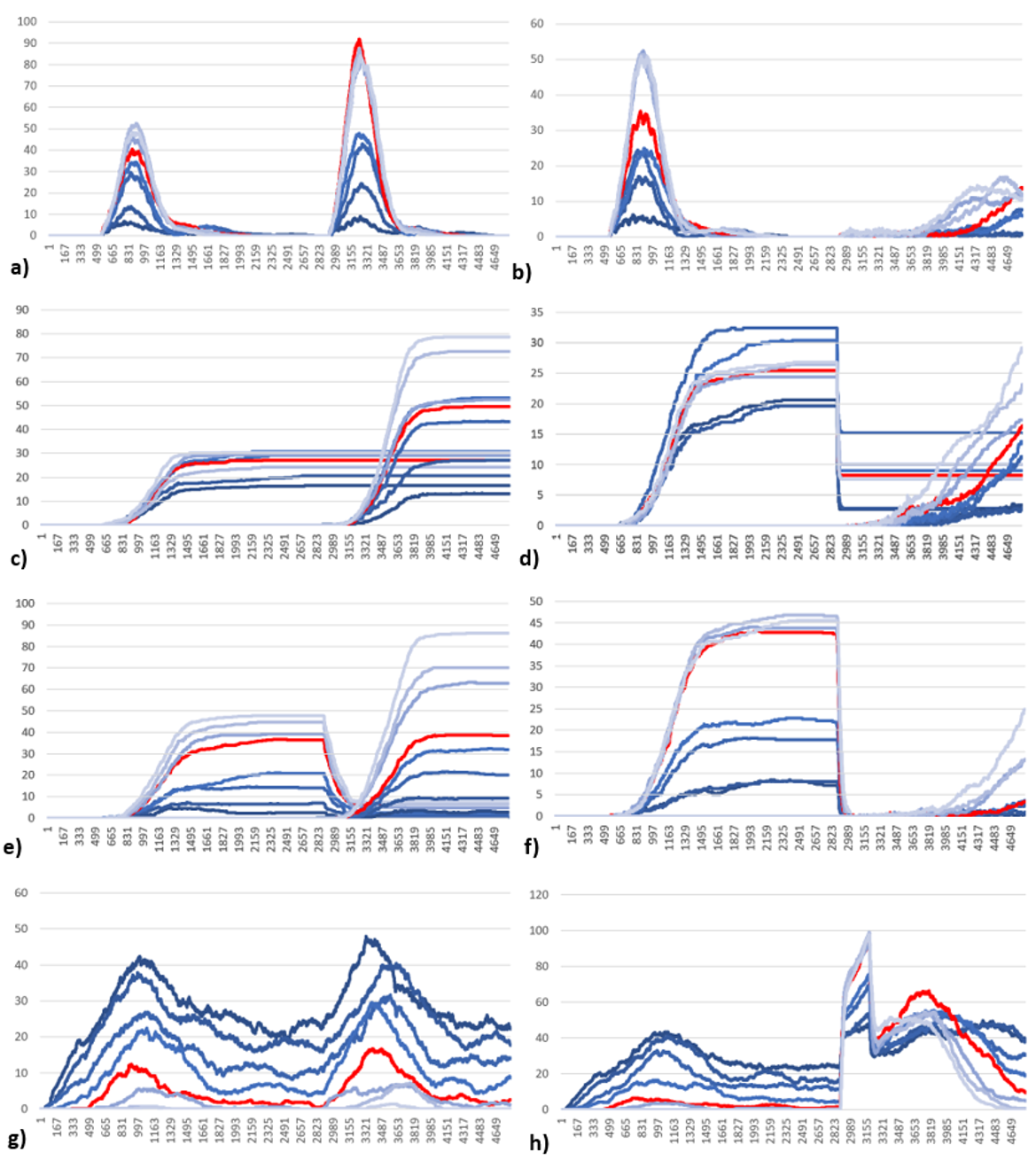

Supplement: S3 Fig — In all panels, the increasingly saturated blue lines represent cell responses to decreasing IL-6 thresholds over which cells undergo differentiation into regulatory B-cells. The red line in each panel represents the median value of incremented thresholds, which was set as the baseline threshold to which to compare. Panels A, C, E, and G demonstrate the responses to the mild antigen stimulus simulations for SLPCs, LLPCs, memory B-cells, and regulatory B-cells, respectively. Panels B, D, F, and H demonstrate the responses to the severe antigen challenge simulations for SLPCs, LLPCs, memory B-cells, and regulatory B-cells, respectively. (TIF) [file pcbi.1011776.s007.tif]
